# Highly Tunable Light Absorber Based on Topological Interface Mode Excitation of Optical Tamm State

**DOI:** 10.3390/s24175772

**Published:** 2024-09-05

**Authors:** Xiangjun Liu, Jingxu Shi, Yixuan Wang, Shiyao Sun, Xiangfu Wang

**Affiliations:** 1 College of Electronic and Optical Engineering & College of Flexible Electronics (Future Technology), Nanjing University of Posts and Telecommunications, Nanjing 210023, China; b21020711@njupt.edu.cn (X.L.); sjx2895275859@outlook.com (J.S.); b21020506@njupt.edu.cn (Y.W.); xjliusummer@gmail.com (S.S.); 2 Yunnan Key Laboratory of Electromagnetic Materials and Devices, Kunming 650091, China; 3 Key Laboratory of Radio Frequency and Micro-Nano Electronics of Jiangsu Province, Nanjing 210023, China

**Keywords:** topological interface modes, optical Tamm state, graphene nanocomposites, optical absorbers, photonic crystals

## Abstract

Optical absorbers based on Tamm plasmon states are known for their simple structure and high operational efficiency. However, these absorbers often have limited absorption channels, and it is challenging to continuously adjust their light absorption rates. Here, we propose a Tamm plasmon state optical absorber composed of a layered stack structure consisting of one-dimensional topological photonic crystals and graphene nano-composite materials. Using the four-by-four transfer matrix method, we investigate the structural relationship of the absorber. Our results reveal that topological interface states (TISs) effectively excite the optical Tamm state (OTS), leading to multiple absorption peaks. This expands the number of absorption channels, with the coupling number of the TIS determining the transmission quality of these channels—a value further adjustable by the period number of the photonic crystals. Tuning the filling factor, refractive index, and thickness of the graphene nano-composite material allows for a wide range of control over the device’s absorption rate, from 0 to 1. Additionally, adjusting the defect layer thickness, incident angle, and Fermi energy enables us to control the absorber’s operational bandwidth and the switching of its absorption effect. This work presents a new approach to expanding the tunability of optoelectronic devices.

## 1. Introduction

Photonic crystals (PCs), which are periodic dielectric structures, have provided opportunities for controlling light–matter interactions due to their exceptional optical properties. One-dimensional photonic crystals (1DPCs) have emerged as the most extensively studied structures due to their simple design and ease of fabrication [1,2,3,4,5]. Over the past few decades, PCs have found applications in various optoelectronic devices, with optical absorbers, as fundamental optoelectronic devices, receiving significant attention [6,7,8,9,10,11]. Optical Tamm states (OTSs) existing on the heterogeneous structure interfaces of PCs are well-known surface waves [12,13,14,15,16]. Unlike traditional surface waves, TE and TM waves can directly excite the OTS without requiring specific incident angles. Hence, numerous absorber devices based on OTSs have been proposed. In 2021, Wu et al. designed a broadband wide-angle absorber based on OTSs at the interface of photonic crystals with a metal layer containing hyperbolic metamaterials, achieving an operational bandwidth of 70° [17]. In 2022, Jie et al. achieved tunable coupling between Tamm plasmon polaritons and Fabry–Pérot cavity modes and accordingly designed a strain sensor [18]. In 2023, Wu et al. proposed utilizing angle-insensitive Tamm plasmon polaritons to promote graphene’s wide-angle and efficient absorption [19]. However, traditional OTS-based absorbers typically consist of heterogeneous structures composed of metal layers and PCs. Due to the high loss of metal layers, their tunability is limited, especially in terms of the adjustability of absorption channels and the absorption rate, restricting the precise control of optical signals. Therefore, designing a new structure to expand the tunability of devices would be highly valuable.

Nano-composite materials with multiple adjustable parameters have significant advantages over devices that can only be adjusted by changing the selection and thickness of metal layer materials. Research has shown that OTSs can also form at the interface between PCs and nano-composite materials [20]. In 2016, Vetrov et al. proposed the existence of OTSs at the interface of PCs with nano-composite materials containing core-shell particles. In 2023, Avdeeva et al. theoretically predicted the possibility of manufacturing polarization-sensitive absorbers based on Tamm plasmon polariton splitting at the interface between metal films and anisotropic nano-composite layers conjugated with PCs [21]. Graphene is not only an important material in optoelectronics but also a crucial filler in composite material applications, possessing excellent thermal, mechanical, and electrical properties compared to traditional nano-composite materials. More importantly, graphene is essentially a semimetal and exhibits certain metallic characteristics under certain conditions. In 2017, Wang et al. achieved tunable terahertz perfect absorbers using graphene’s Tamm surface protons [22], suggesting that the configuration of graphene nano-composite material (GNC)’s 1DPCs may also excite the OTS. Moreover, the topic of adding graphene to the structure of composite materials has been extensively treated experimentally [23,24,25,26].

Recently, it has been demonstrated that a topological interface state (TIS) can appear at the interfaces of two PC regions with opposite band characteristics [27]. A TIS can generate a protected non-scattering transmission at material boundaries due to the topological protection effect, enabling light transmission even in the presence of defects or disturbances. Leveraging its simplicity and excellent light manipulation capabilities, the TIS has been utilized in various optoelectronic devices such as perfect absorbers [28,29] and sensors [30,31]. The topological properties of one-dimensional periodic structures can be characterized by the Zak phase, which describes the photonic band structure and can predict the presence of interface states. The Zak phase is related to the sign of the reflection phase in the 1DPC bandgap and can be easily measured in the optical range [32]. Moreover, the coupling of multiple TISs can generate stable multi-channel light transmission [32,33]. In 2018, Yoichiro et al. reported topologically protected optical Tamm states [34]. Subsequently, in 2020, a study demonstrating the tunable control of light absorption excited by topological interface states was reported [35]. Furthermore, to design an optical absorber with excellent tunability, leveraging the excitation of the OTS at the interface between a photonic crystal (PC) and nanocomposite material by topological interface modes is promising, as the excitation conditions for the OTS are straightforward.

In this study, we propose an optical absorber composed of one-dimensional topological photonic crystals (1DTPCs) and a GNC. Our research demonstrates that altering the number of interfaces between photonic crystals with different band features directly regulates the number of absorption channels, and the absorption quality varies depending on the number of coupled TISs. Not only can the quality factor of absorption peaks be further adjusted by changing the period number of the photonic crystal, but also the absorption rate can be tuned by altering the filling factor, refractive index, and thickness of the nano-composite material. Additionally, changes in the incident angle and the addition of defect layers can adjust the position of absorption peaks while maintaining a high level of absorption. Finally, we discuss the absorption response of devices at different Fermi energies, showing distinct switching characteristics.

## 2. Materials and Methods

**Figure 1:**
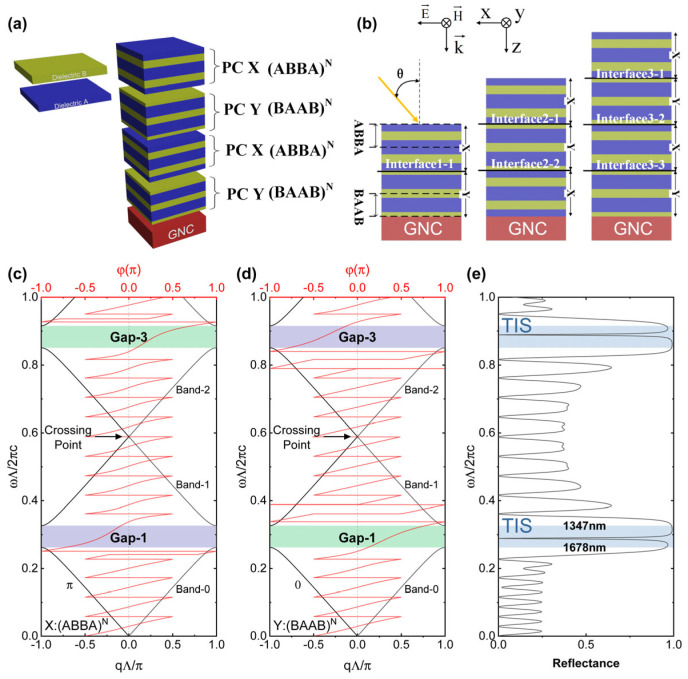
(**a**) The proposed structure model of HTA. (**b**) The cross-sectional structures of HTA with interface numbers of 1 (X|YG), 2 (X|Y|XG), and 3 (X|Y|X|YG). The photonic band structures and reflection phase spectra of PCX and PCY are plotted in (**c**,**d**), respectively, when N = 5. Green bands represent gaps with positive topological properties (φ>0), while purple bands represent gaps with negative topological properties (φ<0). The Zak phase of each band (Band 0, Band 1, and Band 2) is annotated on the left side of the band. (**e**) Reflectance spectra of the X|Y structure under normal incidence condition.

Figure 1a illustrates the alternating stacking of PCX and PCY on a GNC substrate with a thickness of $d_{S}$, forming a highly tunable absorber (HTA). PCX consists of N “ABBA” units, while PCY consists of N “BAAB” units. Notably, PCX and PCY possess symmetry and topological characteristics and are commonly referred to as 1DTPCs. Compared to traditional 1DPCs composed of N “AB” units, 1DTPCs can achieve more stable full-transmission resonant modes under topological protection, even in the case of structural defects or manufacturing errors, making them less susceptible to these disturbances. Therefore, 1DTPCs offer significant advantages in the design of photonic devices that require high levels of stability and robustness against disturbances. The dielectric materials A and B are selected as Ta_2_O_5_ [36] and SiO_2_ [37], respectively, with refractive indices of $n_{A}=2.05,\;n_{B}=1.45$. The thicknesses of layers A and B are set to $d_{A}=91\mathrm{nm}$ and $d_{B}=129\mathrm{nm}$, respectively, to establish suitable photonic bandgaps (PBGs) in the near-infrared region. The growth method of PCs composed of the above materials has been reported in reference [38].

Figure 1b displays the cross-sectional view of the OTS structure, in which the incident surface is chosen in the x-z plane, and the beam is incident on the HTA at an angle θ with the +z-axis. The positions at which various topological interface modes are excited are highlighted, and the corresponding names are provided for identification. 

The following assumptions were made during the modeling process:

1. Each layer of material in the device is considered isotropic;
2. The principal axis of the crystal system coincides with the principal axis of the laboratory system;
3. The incident and exit medium on both sides of the simulated structure is air with a refractive index of 1;
4. The media in the device are assumed to be non-magnetic.

### 2.1. Permittivity of Graphene Nanocomposite Material

The Maxwell–Garnett model is employed to compute the refractive indices of this composite material [39,40,41]. This composite integrates graphene with the host medium, and its dielectric constant is deduced using the specified models as follows:

(1) $$\varepsilon_{\mathrm{GNC}}=\frac{2\varepsilon_{h}V(\varepsilon_{G}-\varepsilon_{h})+\varepsilon_{G}\varepsilon_{h}+2{\varepsilon_{h}}^{2}}{2\varepsilon_{h}+\varepsilon_{G}+V(\varepsilon_{G}-\varepsilon_{h})}$$

where $\varepsilon_{h}={n_{h}}^{2}$ is the dielectric constant of the host medium in the composite, $\varepsilon_{G}$ is the dielectric constant of graphene, and V is the filling factor of the composite. 

The dielectric constant of graphene is described by the following equation [42]:

(2) $$\varepsilon_{G}=1+\frac{i\sigma_{G}}{\omega \varepsilon_{0}d_{G}}$$

where ω is the frequency of the incident light, $\varepsilon_{0}$ is the vacuum dielectric constant, and $d_{G}$ is the thickness of the monolayer graphene, which is set to 0.34 nm here. $\sigma_{G}$ is the electrical conductivity of the graphene, which is composed of the following two factors:

(3) $$\sigma_{\mathrm{intra}}=\frac{ie^{2}}{\pi \hbar^{2}}\frac{\left|E_{f}\right|}{\omega +i\tau^{-1}}$$

(4) $$\sigma_{\mathrm{inter}}=\frac{ie^{2}}{4\pi \hbar }\ln\left(\frac{2|E_{f}|-\left(\omega +i\tau^{-1}\right)\hbar }{2|E_{f}|+\left(\omega +i\tau^{-1}\right)\hbar }\right)$$

where $E_{f}$ is the Fermi energy of graphene, ω is the frequency of incident light, τ is the electronic relaxation time in graphene, $\tau =\mu E_{f}/e{V_{f}}^{2}$, $V_{f}$ is the Fermi velocity in graphene, μ is the carrier mobility in graphene, and ℏ is the approximate Planck constant.

### 2.2. 4 × 4 Transfer Matrix Method (TMM)

Based on Maxwell’s equations, the electric field in the medium satisfies the following equations:

(5) $$\nabla^{2}E+{k_{0}}^{2}\varepsilon E=\nabla (\nabla \cdot E)$$

where E is the electric field and $k_{0}=2\pi /\lambda$ is the wave vector.

We use a 4 × 4 TMM [43] for the above equations in order to obtain the response of the device at different polarized incidence values. The total transfer matrix equation for the device can be written in the following form [43]:

(6) $$\left(\begin{array}{c} A_{s}\\ B_{s}\\ A_{p}\\ B_{p} \end{array}\right)=\left(\begin{array}{cccc} T_{11} & T_{12} & T_{13} & T_{14}\\ T_{21} & T_{22} & T_{23} & T_{24}\\ T_{31} & T_{32} & T_{33} & T_{34}\\ T_{41} & T_{42} & T_{43} & T_{44} \end{array}\right)\left(\begin{array}{c} C_{s}\\ 0\\ C_{p}\\ 0 \end{array}\right)=T\left(\begin{array}{c} C_{s}\\ 0\\ C_{p}\\ 0 \end{array}\right)$$

where T is the total transfer matrix of the system, A, B, and C represent the amplitudes of incident, reflected, and transmitted light, and s, p represent TE and TM polarized waves.

The total transfer matrix for a multilayer system is as follows:

(7) $$T={L_{a}}^{-1}\prod_{n=1}^{N}{\left[T_{np}(d_{n})\right]}^{-1}L_{f}={L_{a}}^{-1}\prod_{n=1}^{N}\left[T_{np}(-d_{n})\right]L_{f}$$

where ${L_{a}}^{-1}$ is the incidence matrix, $L_{f}$ is the exit matrix, and $T_{np}$ is the transfer matrix of the intermediate isotropic layer.

(8) $$T_{np}=\left(\begin{array}{cccc} \cos k_{0}d_{i}q_{i} & 0 & 0 & i\frac{q_{i}}{\varepsilon_{i}}\sin k_{0}d_{i}q_{i}\\ 0 & \cos k_{0}d_{i}q_{i} & -\frac{i}{q_{i}}\sin k_{0}d_{i}q_{i} & 0\\ 0 & -iq_{i}\sin k_{0}d_{i}q_{i} & \cos k_{0}d_{i}q_{i} & 0\\ i\frac{\varepsilon_{i}}{q_{i}}\sin k_{0}d_{i}q_{i} & 0 & 0 & \cos k_{0}d_{i}q_{i} \end{array}\right)$$

where $q_{i}=\sqrt{\varepsilon_{i}-{k_{x}}^{2}}=\sqrt{{n_{i}}^{2}-{n_{a}}^{2}\sin^{2}\theta }$, $d_{i}$, $n_{i}$ is the thickness and refractive index of the intermediate layers.

The reflection and refraction coefficients of the device can be derived directly from the following equations:

(9) $$r_{\mathrm{TM}}=\frac{T_{11}T_{43}-T_{41}T_{13}}{T_{11}T_{33}-T_{13}T_{31}}\;,\;t_{\mathrm{TM}}=\frac{T_{11}}{T_{11}T_{33}-T_{13}T_{31}}$$

(10) $$r_{\mathrm{TE}}=\frac{T_{21}T_{33}-T_{23}T_{31}}{T_{11}T_{33}-T_{13}T_{31}}\;,\;t_{\mathrm{TE}}=\frac{T_{33}}{T_{11}T_{33}-T_{13}T_{31}}$$

So, the absorptivity of the whole device can be described as follows:

(11) $$A_{\mathrm{TM}}=1-R_{\mathrm{TM}}-T_{\mathrm{TM}}$$

(12) $$A_{\mathrm{TE}}=1-R_{\mathrm{TE}}-T_{\mathrm{TE}}$$

where $R_{\mathrm{TM}}=|r_{\mathrm{TM}}{|}^{2}$, $R_{\mathrm{TE}}=|r_{\mathrm{TE}}{|}^{2}$, $T_{\mathrm{TM}}=|t_{\mathrm{TM}}{|}^{2}$, $T_{\mathrm{TE}}=|t_{\mathrm{TE}}{|}^{2}$.

### 2.3. Calculation of Normalized Electric Field Intensity Based on TMM

With $z_{0}$ represent the coordinate where the optical wave enters the HTA, the interfaces with two different media that it passes through are located at $z_{1}$, $z_{2}$, …, $z_{i}$, respectively. The following is the expression of the distribution of the electromagnetic field within the layer as a function of z:

(13) $$\Psi \left(z\right)={\left(\begin{array}{cccc} E_{x} & E_{y} & H_{x} & H_{y} \end{array}\right)}^{T}\left(z\right)$$

Matrices $T_{np}$ can achieve the following matrix transformations [43]:

(14) $$\Psi \left(z_{n}\right)=T_{np}\left(z_{n}-z_{n-1}\right)\Psi \left(z_{n-1}\right)$$

The electromagnetic field distribution of the plane with coordinates z within the ith layer can be computed as follows:

(15) $$\Psi \left(z\right)=T_{ip}(z-z_{i-1})\cdots T_{2p}(z_{2}-z_{1})T_{1p}(z_{1}-z_{0})\Psi \left(z_{0}\right)$$

(16) $$\Psi \left(z_{0}\right)=T_{E}\Psi \left(z\right)$$

where $T_{E}\left(z\right)$ is a function of the coordinate z given by the following:

(17) $$T_{E}\left(z\right)=\prod_{n=1}^{i-1}T_{np}(-d_{n})T_{ip}(z_{i-1}-z)$$

where $d_{n}=z_{n}-z_{n-1}$. Equation (16) evolves to the following to compute Ψ(z):

(18) $$\Psi \left(z\right)={T_{E}}^{-1}\Psi \left(z_{0}\right)$$

The solution for $\Psi \left(z_{0}\right)$ refers to the case of single-polarization wave incidence. The normalization of the electric field at $z_{0}$ to 1, under TM polarization, can be expressed as follows:

(19) $$\Psi \left(z_{0}\right)=\left(\begin{array}{c} E_{x0}\\ 0\\ 0\\ H_{y0} \end{array}\right)=\left(\begin{array}{c} 1\\ 0\\ 0\\ \frac{1+r_{p}}{1-r_{p}} \end{array}\right)=\left(\begin{array}{c} 1\\ 0\\ 0\\ \frac{T_{33}+T_{43}}{T_{33}-T_{43}} \end{array}\right)\;(\mathrm{TM}\;\mathrm{waves})$$

Thus, relying on Equation (19), the calculated $E_{x}$ is equal to the normalized electric field $E_{x}/E_{x0}$.

## 3. Results and Discussion

Based on the TMM [43], this study developed relevant code using MATLAB to compute the optical effects of the proposed structure. Figure 1c,d show the band structures of the “ABBA”- and “BAAB”-type PCs represented by PCX and PCY, respectively. One-dimensional systems with inversion symmetry always have two inversion centers. As the origins of PCX and PCY as inversion centers differ, the Zak phase of Band-0 will be quantized as π and zero, respectively. The phase sign of the nth gap is described as follows [27]:

(20) $$\mathrm{sgn}\left[\varphi_{n}\right]={\left(-1\right)}^{n}{\left(-1\right)}^{l}\exp\left(i\sum_{m=0}^{n-1}\theta_{m}^{Zak}\right)$$

where l is the total number of crossing points before the nth gap, and $\theta_{m}^{Zak}=0$ or π is the Zak phase of the mth band (m≠0). 

Therefore, PCX and PCY will have opposite phase signs in the first and third gaps, which will excite the TIS when combined and produce an efficient transmission peak (reflection valley) within the PBG, as shown in Figure 1e. The TISs between PCs efficiently transfer energy backward, which will excite OTS modes near the incident surface of the GNC. For the proposed HTA structure in this paper, we focused on the 1300 nm to 1700 nm wavelength band corresponding to Gap-1.

Based on the theory of one-dimensional topological photonics, when the sum of the surface impedances of two semi-infinite PCs equals zero, the TIS can be excited at their central interface [27]. An important criterion for finding the occurrence of the TIS on the PC surface is that the sum of the surface impedances before and after the interface is equal to zero. The equivalent impedance of an optical structure can be calculated by the following equation [44]:

(21) $$Z=\sqrt{\frac{(1+r^{2})-t^{2}}{(1-r^{2})-t^{2}}}$$

where r and t are the reflection and transmission coefficients of the structure, respectively. Under the assumption proposed in this paper and for vertical incidence conditions, TM waves and TE waves will have the same optical response, so r and t can be described using Equation (9). Within the bandgap, the reflection effect of the PC is significant, and t can be ignored. The complex reflection coefficient r with a magnitude of 1 can be written as $r=e^{i\varphi }$, where φ is the reflection phase. Therefore, the condition that the sum of the impedances before (front) and after (back) the interface equals zero ($Z_{front}+Z_{back}=0$) corresponds to the following system of equations:

(22) $$\frac{1+\cos(2\varphi_{front})}{1-\cos(2\varphi_{front})}=-\frac{1+\cos(2\varphi_{back})}{1-\cos(2\varphi_{back})}\left(\mathrm{Real}\;\mathrm{part}\right)$$

(23) $$\frac{\sin(2\varphi_{front})}{1-\cos(2\varphi_{front})}=-\frac{\sin(2\varphi_{back})}{1-\cos(2\varphi_{back})}\left(\mathrm{Imaginary}\;\mathrm{part}\right)$$

These correspond to the following unique solution:

(24) $$\varphi_{front}+\varphi_{back}=0$$

Under the condition of good reflection effects on both sides, the TIS will appear at frequencies at which the sum of the reflection phases on both sides equals zero. Figure 1e illustrates the situation of a single TIS, and increasing the number of interfaces will lead to the coupling of multiple TISs [32,33]. 

**Figure 2:**
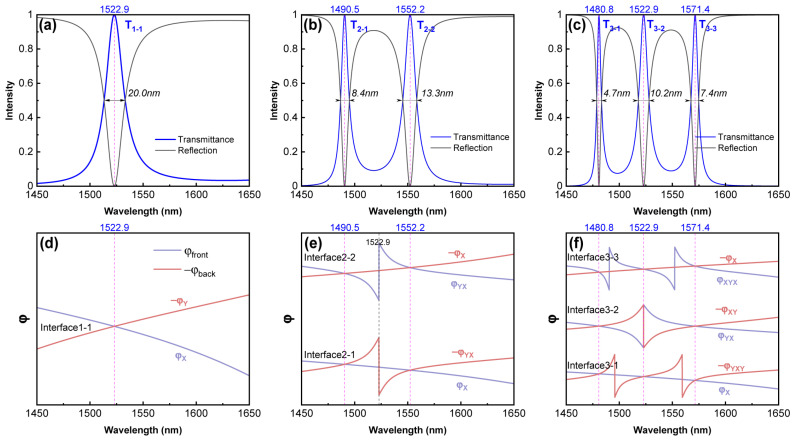
Transmittance and reflection spectra of topological interface structures with interface numbers of 1 (X|Y), 2 (X|Y|X) and 3 (X|Y|X|Y) under normal incident light with N = 5 are plotted in (**a**–**c**), respectively. The distribution of reflection phase on both sides of each interface within the structure is depicted in (**d**–**f**). Black font is used to label the full width at half maximum (FWHM) of transmittance peaks or interface names, while magenta dashed lines indicate the wavelengths corresponding to each peak. The blue/purple and brown/red lines represent the $\varphi_{front}$ and $-\varphi_{back}$, respectively, where $\varphi_{front}$ denotes the phase before the interface and $\varphi_{back}$ denotes the phase after the interface.

Figure 2a–c show the transmission spectra of multi-layer PCs’ structures with different numbers of interfaces, in which the substrate-free HTA is referred to as the topological interface structure. The positions of transmission peaks are indicated by purple dashed lines, labeled as T with different subscripts. TISs typically manifest as special modes capable of capturing and transmitting energy at the interface. Their existence with topological protection ensures stability and efficiency, resulting in transmission peaks with peak values greater than 0.99 across all spectra, as shown in Figure 2a–c. Multiple interfaces successfully induce the phenomenon of multiple TISs, and the number of transmission peaks is directly correlated with the number of interfaces between the PCs with distinct band characteristics. Strong coupling between TISs effectively enhances the quality of resonance states within the structure, and as the number of interfaces increases, the full width at half maximum (FWHM) of the transmission peaks generally decreases. In addition, since there is no light loss due to the lossy material, each transmission peak will correspond well to the reflection valley within the PBG.

Figure 2d–f illustrate the variation in the reflection phase on both sides of each interface. The phase $\varphi_{back}$ is negated, making the intersection points in the figure correspond to the solution of Equation (24). When an interface has both PCX and PCY, an phase shift of approximately π can be observed. Comparing the purple dashed line in Figure 2d with the black dashed line in Figure 2e, these phase shifts occur due to both the real and imaginary parts of the reflection coefficient crossing zero, which will occur at positions where the structure on this side generates a perfect transmission. Thus, for the purple dashed line in Figure 2f positioned in the middle, when the incident wavelength is 1522.9 nm, the incident light can pass completely through Interface3-2 without reflection, and no TISs are formed at Interface3-2. The T_3-2_ is therefore a result of the coupling of two TISs, with its FWHM significantly larger than the transmission peaks on both sides. Additionally, all interfaces within the structure are well aligned with the wavelengths that induce transmission peaks, forming strong resonance between multiple induced TISs as shown in Equation (24).

The Fermi energy of graphene material is typically set to 0.2 eV. A doping concentration of V=0.05 and a substrate of Si [45] ($n_{h}=3.47$) were used. Following the introduction of a GNC layer with a thickness of 4μm behind the topological interface structure, an HTA structure was formed, as shown in Figure 1a. Building upon the efficient transmission demonstrated in Figure 2, the GNC layer at the end of the structure can efficiently absorb light based on the principles of OTS when excited by TIS modes.

**Figure 3:**
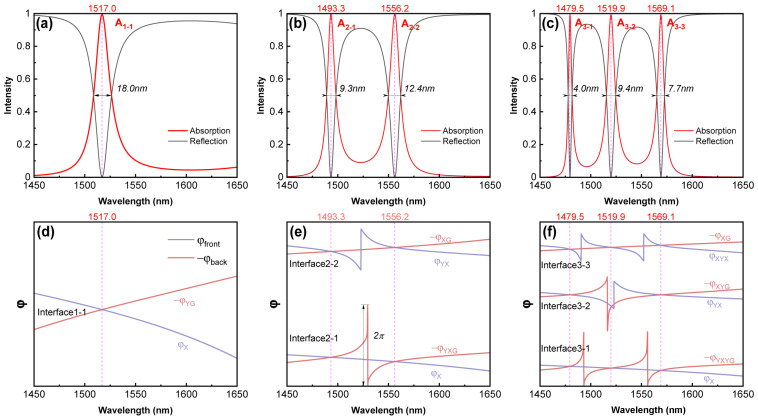
Absorption spectra of HTA with interface numbers of 1 (X|YG), 2 (X|Y|XG), and 3 (X|Y|X|YG) are plotted in (**a**–**c**), respectively. The distribution of reflection phase on both sides of the interface is depicted in (**d**–**f**), respectively. Black font is used to label the FWHM of absorption peaks or interface names, while magenta dashed lines indicate the wavelengths corresponding to each peak. Blue/purple and brown/red lines represent the regions before and after the interface, respectively. Here, $d_{S}$ = 4 μm and N = 5.

Figure 3a–c display the absorption spectra of the HTA structure. Each absorption peak corresponds well with the transmission peaks shown in Figure 2 and exhibits identical interface state coupling conditions with peak values exceeding 0.99, achieving perfect absorption. Due to the stability conferred by the 1DPCs’ topological properties, the addition of the GNC layer only introduces minor shifts in the wavelength distribution and the FWHM of the absorption peaks shown in Figure 3, compared to the transmission peaks in Figure 2.

Figure 3d–f illustrate the changes in the reflection phase on each side of the interface in the HTA structure. When the rear side of the interface contains both the interface and GNC simultaneously, $\varphi_{back}$ will undergo a 2π phase shift, as the intersection points of the real and imaginary parts of $r_{back}$ diverge significantly from zero. For A_3-2_, Interface3-2 in the HTA still fails to form a TIS because the reflection responses on both sides of the interface are too weak. Additionally, each interface in the HTA structure satisfies the phase-matching condition at the peak, efficiently transferring energy toward the GNC’s direction without a reflection. Overall, after adding the GNC layer, the absorption effect effectively replaces the original transmission effect. As observed in Figure 2, there is a notable symmetry between the absorption and reflection spectra. Our numerical validation confirms that all subsequent analyses of the absorption peaks in the HTA can be equivalently mapped to the reflection troughs. This provides a more direct and measurable parameter for sensor applications. 

**Figure 4:**
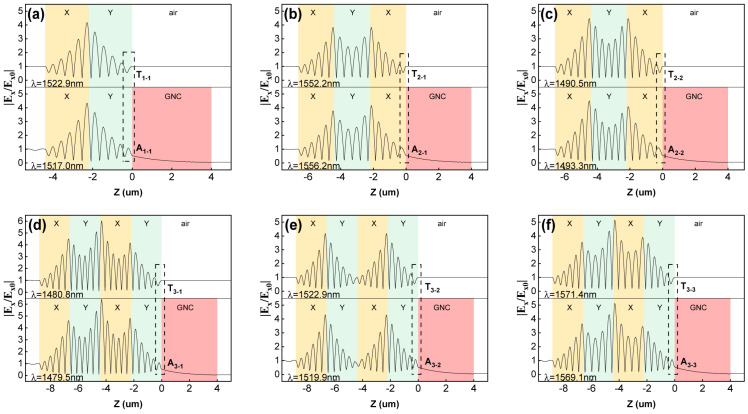
The influence of GNC layer on the normalized electric field intensity inside the HTA structure. The upper halves of each image show the normalized electric field distribution inside the structure corresponding to T peaks with different subscripts in the absence of GNC layer. After adding a GNC layer with $d_{S}$ = 4 μm, the normalized electric field distribution inside the structure corresponding to peak A is depicted below the T peaks with the same subscript. The field intensity spectra under each peak of HTA with interface numbers of 1, 2, and 3 are plotted in (**a**), (**b**,**c**), (**d**–**f**), respectively. Other parameters are consistent with Figure 3.

To investigate the energy transfer within the structure, Figure 4 illustrates the normalized electric field intensity distribution inside the HTA structure. As a one-dimensional device, the zero point of the Z-coordinate is chosen at the exit interface of the topological interface structure. The yellow, cyan, and red regions in Figure 4 represent PCX, PCY, and the GNC in the HTA, respectively, while the white area represents the air outside the structure.

In the upper part of Figure 4a, the topological interface structure of a single interface (X|Y) is displayed. The peak of the normalized electric field intensity is confined near the interface between PCX and PCY, indicating that the excitation of TIS modes effectively promotes the operation of resonant modes within the structure, allowing for photon accumulation around this interface and efficient excitation for backward transmission. In the lower part of Figure 4a, a layer of GNC with a thickness of 4μm is added. Due to the mild optical properties of the GNC and its thickness not significantly exceeding that of the topological interface structure, there is no significant change in the distribution of normalized electric fields within the topological interface structure, further confirming that the addition of GNC does not disrupt the formation of TIS at the interface between PCX and PCY. However, due to the introduction of a heterostructure, a peak appears at the interface between the PC and GNC, as indicated by the black dashed box in the figure, which is considered an external manifestation of the OTS excited by the TIS. Based on the good light absorption characteristics of the GNC, when photons are excited into the GNC by TISs, they are gradually absorbed, eventually approaching zero, consistent with the phenomenon of perfect absorption.

As the number of interfaces within the structure increases, peaks in the electric field intensity near the excited interface states remain evident. When the introduced number of interfaces reaches two, the electric field intensity distributions near the two interfaces become quite similar. Corresponding to T3-2 and A3-2, in Figure 4e, Interface3-2 exhibits no photon localization phenomenon, consistent with our analysis in Figure 2 and Figure 3. Additionally, in Figure 4d,f, the normalized electric field intensity near Interface3-2 is significantly higher than that near the other two interfaces. The distribution of electric fields within the topological interface structure exhibits a remarkable symmetry, owing to the high similarity between PCX and PCY and the stability of the topological structure. Furthermore, different numbers of TISs can all excite the generation of the OTS at the interface between the PC and GNC, resulting in a peak in the electric field intensity in the region before this interface. In Figure 4, the normalized electric field intensity around the interface in Figure 4d is the highest, indicating that A_3-1_ corresponds to the best performance.

To assess the performance of multiple absorption peaks in the HTA, the quality factor Q is derived from the following equation [46]:

(25) $$Q=\frac{f_{T}}{FWHM}$$

where $f_{T}$ is the resonance frequency, and FWHM is the full width at half maximum of the absorption peak.

**Figure 5:**
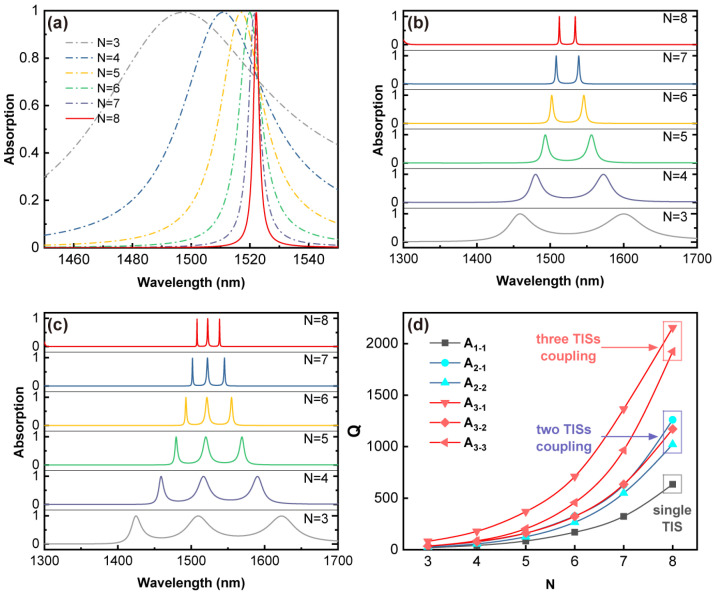
The absorption spectra of HTA with interface numbers of 1 (X|YG), 2 (X|Y|XG), and 3 (X|Y|X|YG) are plotted as a function of the period number N in (**a**–**c**), respectively. (**d**) Quality factor of absorption peaks of HTA as a function of N. Other parameters are consistent with Figure 3.

For an HTA structure with any number of interfaces, the quality factor of the absorption peak can be adjusted over a wide range by changing the number of periods of the PC. As shown in Figure 5a, as the number of periods N of the PC increases, the value of absorption peaks remains essentially unchanged, and the quality factor is significantly increased. Figure 5d numerically shows that the quality factor of A_1-1_ increases from 19.7 to 634 as the period number increases. At this point, the change in the position of the absorption peak is due to the effective reduction in the influence of the nano-composite material on $\varphi_{back}$ with the increase in the number of periods of the PC. Figure 5b,c, respectively, show the absorption spectra for the double interface (X|Y|XG) and triple interface (X|Y|X|YG) cases. As the number of periods N of the PC increases, their absorption peak values remain basically unchanged, and the quality factors are significantly increased. 

Figure 5d plots the function graph of the quality factor of all absorption peaks as a function of the period number N. Based on the numerical values, three categories of absorption peaks can be classified: those excited by a single TIS with the highest quality factor reaching around 600 (A_1-1_), those excited by the coupling of two TISs with the highest quality factor reaching around 1200 (A_2-1_, A_2-2_, and A_3-2_), and those excited by the coupling of three TISs with the highest quality factor reaching around 2000 (A_3-1_ and A_3-3_). Among them, the quality factor of A_3-1_ is significantly larger than that of A_3-3_, which benefits from the highest internal normalized electric field intensity at the frequency corresponding to A_3-1_. The results in Figure 5d further demonstrate that the coupling of multiple TISs can effectively improve the quality of energy transfer. In addition, strong coupling between multiple TISs can lead to Rabi-like splitting, resulting in energy separation between different resonance states. Furthermore, the absorption induced by the OTS excited by TISs also correspondingly leads to splitting, manifested as wavelength differences between absorption peaks in Figure 5b,c. Figure 5b,c demonstrates that increasing N can effectively reduce the energy separation between multiple absorption peaks, consistent with the results in the reference [32]. In general, the design of the HTA can achieve the multi-channel absorption with high values of Q. Based on the multi-dimensional tunability described in the following paper, it is expected to bring new implications for the design of high-resolution multifunctional sensors.

**Figure 6:**
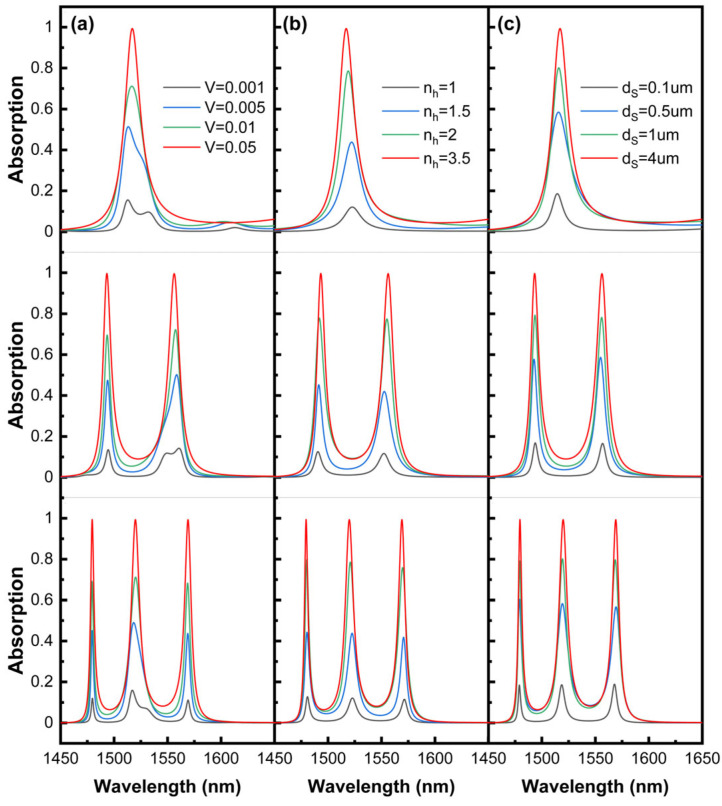
The effects of filling factor V, host medium refractive index $n_{h}$, and thickness $d_{S}$ of nanocomposite materials on the absorption spectra of HTA are plotted in (**a**–**c**), respectively. Other parameters are consistent with Figure 3.

Figure 6 illustrates the adjustment of the filling factor V of the nanocomposite material (Figure 6a), the refractive index $n_{h}$ of the host medium (Figure 6b), and the thickness $d_{S}$ (Figure 6c) on the size of the absorption peaks in the HTA. The increase in the refractive index of the matrix and the filling factor enhances the imaginary part of the dielectric constant of the nanocomposite material, which determines the absorption effect of the GNC on photons. They, along with $d_{S}$, alter the intensity of the absorption peaks because they limit whether the photons have sufficient paths for the loss. Meanwhile, since the GNC basically does not affect the phase conditions at the interfaces, they bring about minimal shifts in the position of the absorption peaks. Overall, by individually adjusting any one of the parameters V, $n_{h}$, and $d_{S}$, it is possible to achieve the continuous control of the absorption effect of the HTA from zero to one. The HTA will have perfect absorption effects when approaching the values in Figure 3: V = 0.05, $n_{h}$ = 3.47, and $d_{S}$ = 4 μm. The high tunability of nano-composite materials provides a novel approach for flexibly controlling absorption intensity.

**Figure 7:**
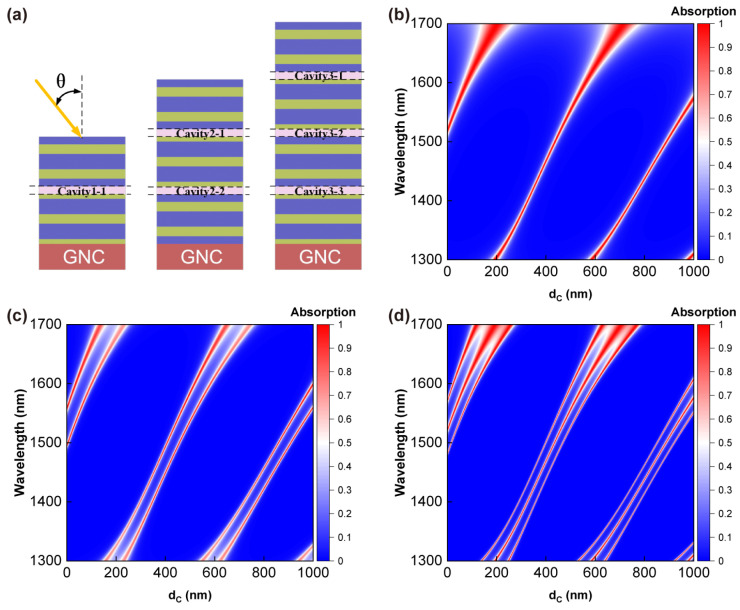
Absorption effects of HTA as a function of defect layer thickness when introducing defect layers at the interfaces. (**a**) Schematic diagram of HTA structure with cavities. (**b**–**d**) display the two-dimensional absorption spectra of HTA with cavity numbers of 1, 2, and 3, respectively. Other parameters are consistent with Figure 3.

For tunable absorptive devices, tuning the resonance position is also crucial. By adding defect layers of the same thickness to each interface of the HTA structure, they possess properties similar to the original interfaces and avoid disrupting the coupling between multiple resonance states. These defect layers are considered as a series of independent Fabry–Perot resonant cavities. Figure 7a illustrates the schematic diagram of the HTA structure with cavities. These cavities are named similarly to the interfaces and labeled on the diagram. They possess similar phase-matching conditions to Equation (24) [28]:

(26) $$\varphi_{front}+\varphi_{cavity}+\varphi_{back}=m*2\pi \;,\;m=0,1,2,\ldots$$

where $\varphi_{cavity}=2\pi *2n_{c}d_{c}/\lambda$ represents the phase delay inside the cavity. $n_{c}$ and $d_{c}$ represent the refractive index and thickness of the cavity, respectively, with $n_{c}=1.66$ (Al_2_O_3_ [47]). m is the order of resonance, which takes natural number values.

Figure 7b illustrates the two-dimensional absorption plot of the HTA with a single cavity, where the absorption peak corresponding to the resonance order m=0 gradually redshifts as the thickness of the defect layer increases from 0 to 200 nm, until it reaches the edge of the PBG and overlaps with the absorption effect outside the PBG. Meanwhile, a new absorption peak corresponding to m=1 appears near 1300 nm, which also redshifts with the increase in $d_{c}$. Therefore, absorption peaks with different resonance orders can flexibly appear within the PBG. Figure 7c,d illustrate the two-dimensional absorption plots of the HTA with two and three cavities, respectively, which similarly apply to the conclusions mentioned earlier and maintain good absorption effects with peak values exceeding 90%.

**Figure 8:**
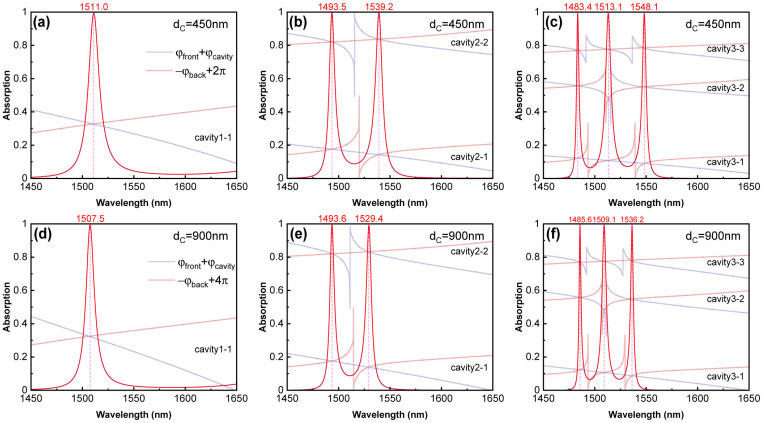
Absorption spectra and phase-matching conditions of HTA with interface numbers of 1 (X|YG), 2 (X|Y|XG), and 3 (X|Y|X|YG) when the defect layer thicknesses are 450 nm and 900 nm. The absorption spectra corresponding to $d_{c}$ = 450 nm are depicted in (**a**–**c**), while those corresponding to $d_{c}$ = 900 nm are shown in (**d**–**f**). Other parameters are consistent with Figure 3.

After the resonant interfaces are replaced by cavities, the resonant conditions inside the cavities still serve as a good basis for determining the position of absorption peaks. Figure 8a–c and Figure 8d–f describe the matching between the absorption spectra of the HTA and the resonance conditions when $d_{c}$ is set to 450 nm and 900 nm, respectively. The red lines in the figures represent absorption curves, and the red font above the images is used to mark the positions of the absorption peaks. When comparing the positions of the absorption peaks, increasing the thickness of the defect layer reduces the linewidth of the absorption peaks and the energy splitting between multiple absorption peaks. When the thickness of the defect layer is 450 nm, the resonance condition of the cavity corresponds to m=1. Therefore, Equation (26) is rewritten as $\varphi_{front}+\varphi_{cavity}=-\varphi_{back}+2\pi$, and the contents of both sides of this equation are drawn in blue/purple and brown/red lines in Figure 8a–c. Similarly, the contents of both sides of $\varphi_{front}+\varphi_{cavity}=-\varphi_{back}+4\pi$ are depicted in Figure 8d–f. Each image in Figure 8 corresponds well with the plots in Figure 3, exhibiting identical phase-matching conditions and the same number of TIS couplings. It can be observed that the introduction of defect layers with the same thickness does not disrupt the operation of the absorber; each absorption peak perfectly conforms to the conditions of Equation (26) and achieves perfect absorption. 

**Figure 9:**
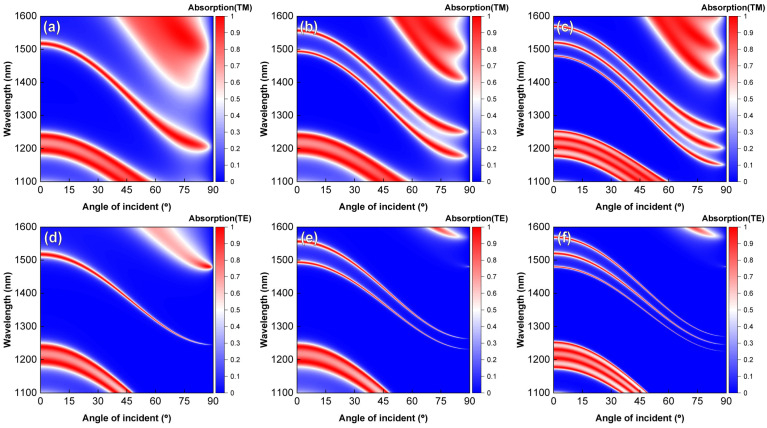
Interface numbers of 1 (X|YG), 2 (X|Y|XG), and 3 (X|Y|X|YG) for HTA regarding the two-dimensional absorption plots of TM/TE polarized waves with respect to incident angles and wavelengths. Absorption spectra under TM polarization are depicted in (**a**–**c**), while those under TE polarization are shown in (**d**–**f**). Other parameters are consistent with Figure 3.

Figure 9 depicts the two-dimensional absorption spectra of three HTA structures with different angles of the TM and TE polarized incidence. Figure 9a–c illustrate the absorption spectra of the device under different angles of TM polarization, showing a minimal shift in the absorption peak positions from 0° to 20°. However, as the incident angle gradually increases to 80°, the absorption peaks exhibit a blue shift of approximately 300 nm. This phenomenon arises because, as the incident angle increases, the resonant wavelength needs to blueshift to satisfy the phase-matching condition due to the increase in θ. At this point, the absorption width extends slightly, while maintaining a peak absorption of around 95%, benefiting from the excitation of the OTS without a specific incident angle requirement.

Figure 9d–f display the absorption spectra of the device under different angles of TE polarization. A comparison between the two sets of figures reveals that under TE polarization, the blueshift of absorption peaks is significantly smaller than that under TM polarization. Moreover, as the incident angle increases, the absorption peaks of the device under TE polarization gradually narrow, and the absorption rate decreases, serving as a potential method for distinguishing between TE and TM polarization.

**Figure 10:**
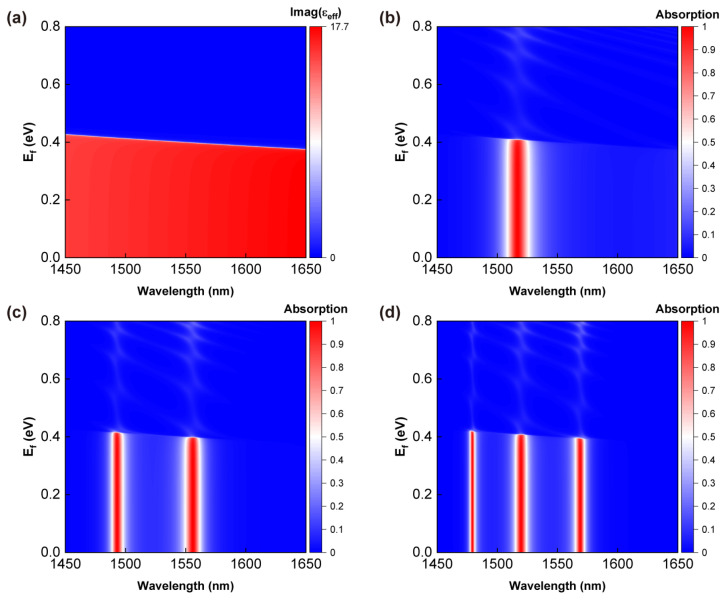
Tuning effect of $E_{f}$ on the absorption of HTA with graphene materials. (**a**) Influence of $E_{f}$ on the imaginary part of the dielectric constant of graphene materials. The effects of $E_{f}$ on the absorption of HTA with 1 (X|YG), 2 (X|Y|XG), and 3 (X|Y|X|YG) interfaces are depicted in (**b**–**d**), respectively. Other parameters are consistent with Figure 3.

As the only loss source, by adjusting the Fermi energy of graphene [42], the absorption of light by the HTA will exhibit a switching effect. For graphene, the Dirac point is typically considered as the “zero” value of the Fermi energy, meaning that at this point, the energy difference between the conduction and valence bands is zero. The Fermi level of graphene is usually defined relative to this Dirac point. Figure 10a describes the variation in the imaginary part of the dielectric constant of graphene nanocomposites with the Fermi energy and wavelength. The remaining figures show the absorption spectra of the HTA structure with N = 7 as a function of the Fermi energy and wavelength. As depicted in Figure 10b–d, when the Fermi energy is less than 0.4 eV, the absorption peak positions and magnitudes for the three structures remain unchanged. However, when the Fermi energy exceeds 0.4 eV, the absorption peaks rapidly decrease to the extent of almost disappearing. This observation aligns well with the results shown in Figure 10a. For Fermi energies less than 0.4 eV, the imaginary part approaches approximately 18, indicating a strong light absorption capability, whereas for Fermi energy levels greater than 0.4 eV, the imaginary part tends to be closer to 0, indicating minimal light–material interaction. This further validates that the absorption of the device is indeed induced by the graphene nanocomposites. This characteristic provides a new perspective on the design of multi-band optical switches.

## 4. Conclusions

This paper utilizes topological interface modes excited in one-dimensional photonic crystals to generate stable light absorption on the surface of nanocomposite materials. The strong coupling between multiple topological interface states is jointly determined by the interfaces of photonic crystals with different band characteristics, significantly affecting the number and quality factor of absorption peaks. Furthermore, by adjusting the period number of the photonic crystal, a wide range of adjustments in the quality factor of the absorption peaks can be achieved. The filling factor, refractive index of the matrix, and thickness of graphene nanocomposites serve as crucial control parameters for the peak absorption rate of the device, allowing for an almost complete adjustment of the absorption rate from 0 to nearly 1. Introducing defect layers and altering the angle of light incidence can achieve redshift and blueshift adjustments of the absorption peaks, respectively. Lastly, the modulation of the graphene Fermi energy results in the device exhibiting characteristics ranging from single-channel to multi-channel optical switching. Our work realizes a highly tunable light absorber, which is anticipated to significantly impact the design of high-resolution multifunctional sensors, optical switches, and other optoelectronic devices.

## Data Availability

Data are contained within the article.
